# Association among handgrip strength, body mass index and decline in cognitive function among the elderly women

**DOI:** 10.1186/s12877-018-0918-9

**Published:** 2018-09-24

**Authors:** Su-min Jeong, Seulggie Choi, Kyuwoong Kim, Sung Min Kim, Sujin Kim, Sang Min Park

**Affiliations:** 1 Department of Family Medicine, Seoul National University Hospital, 101 Daehak-ro, Jongno-gu, Seoul, South Korea; 2 Department of Biomedical Sciences, Seoul National University Graduate School, 101 Daehak-ro, Jongno-gu, Seoul, South Korea; 3 Korea Institute for Health and Social Affairs, Sejong, South Korea

**Keywords:** Handgrip strength, Obesity, Cognitive function

## Abstract

**Background:**

The association between handgrip strength combined with body mass index (BMI) and cognitive impairment has not been thoroughly examined. We aimed to investigate whether the relationship between handgrip strength and risk of cognitive impairment is altered by the presence of obesity in older women.

**Methods:**

A total of 544 older women aged over 65 years without cognitive impairment from the Korean Longitudinal Study of Aging (KLoSA) were included in the study. Handgrip strength was classified in a binary manner (weak or strong) or in tertiles and obesity was defined as a BMI ≥ 25 kg/m^2^, in accordance with the Asia-Pacific World Health Organization criteria. Incident cognitive impairment was defined as a Korean Mini-mental State Examination (K-MMSE) score of less than 24 after eight years of follow-up.

**Results:**

Strong handgrip strength was associated with reduced likelihood of developing cognitive impairment compared to weak handgrip strength in obese women (adjusted odds ratio, aOR 0.23, 95% confidence interval, CI 0.08–0.66). The highest tertile of handgrip strength was associated with reduced risk of incident cognitive impairment (aOR 0.16, 95% CI 0.04–0.70), compared to the lowest tertile of handgrip strength in obese women, with a significant linear trend (*p* for trend = 0.016). Furthermore, the highest tertile of handgrip strength was significantly associated with smaller decline in K-MMSE scores compared to the lowest tertile of handgrip strength in obese women (*p* value = 0.009). There was no association between handgrip strength and incident cognitive impairment in non-obese women.

**Conclusions:**

Strong handgrip strength was associated with reduced risk of cognitive impairment among obese women, but not in non-obese women. Handgrip strength may be a simple and useful marker for predicting future cognitive impairment among obese women.

**Electronic supplementary material:**

The online version of this article (10.1186/s12877-018-0918-9) contains supplementary material, which is available to authorized users.

## Background

Cognitive decline may lead to mild cognitive impairment or dementia among older adults. A recent study has shown that the conversion rate to cognitive impairment is 6% per person-year among healthy adults [1]. Therefore, identifying and managing modifiable risk factors for cognitive decline are imperative. Previously, body mass index (BMI) and weight change has been shown to be associated with cognitive decline [2, 3]. A recent meta-analysis reported that being underweight, overweight, and obese in mid-life were associated with increased risk of dementia [4]. However, in other studies, overweight in late-life was associated with lower risk of dementia [5]. In addition, decline in BMI was associated with reduced memory function.

The protective effect of high BMI on the risk of dementia may be affected by handgrip strength, a surrogate marker for muscle strength. Handgrip strength, which is positively correlated with BMI, is a valid and representative measure of muscle strength that reflects total power from the upper limb muscles [6]. Previous studies have shown that handgrip strength is associated with mortality [7, 8], cardiovascular disease [9, 10] and cognitive function [11–13]. In terms of the temporal relationship between handgrip strength and cognitive function, many studies reported that cognitive decline preceded handgrip strength weakness [11, 14]. Moreover, handgrip strength could be a predictive value for cognitive decline [15]. Strong handgrip strength may imply that the intact neuromuscular integrity is associated with reduced risk of dementia and cognitive decline. Although handgrip strength differs according to BMI [16], most previous studies have simply adjusted for height, weight [11] or BMI [12, 13].

Therefore, we hypothesized that different associations may exist between handgrip strength and cognitive strength according to obesity. This study aimed to investigate whether the relationship between handgrip strength and risk of cognitive impairment is altered by the presence of obesity in older women.

## Methods

### Study population

**Fig. 1:**
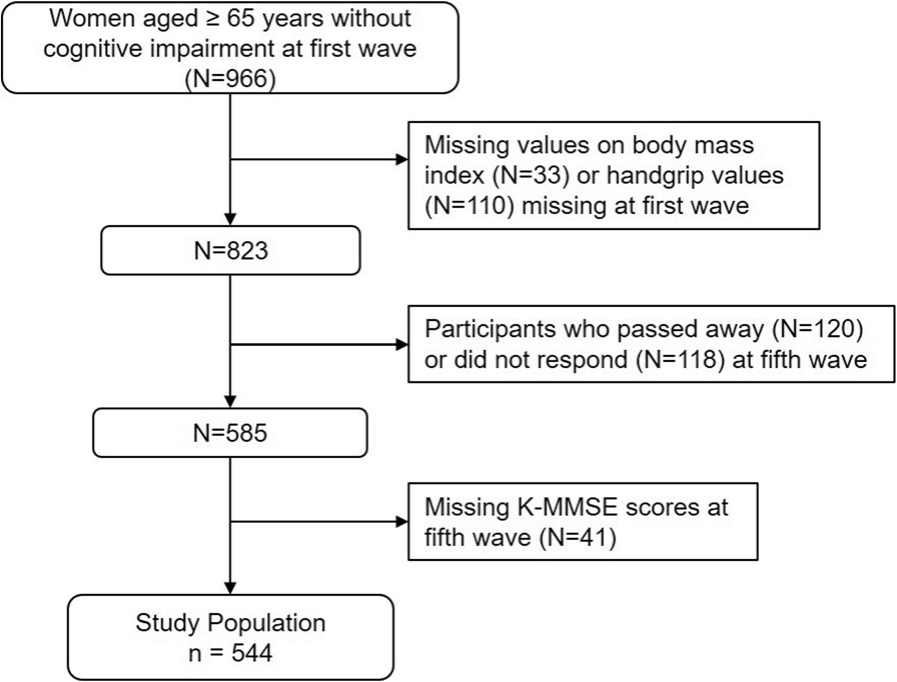
Flow diagram of the study population. Acronyms: K-MMSE, Korea Mini-mental State Examination

The study population was derived from a nationwide panel survey on individuals over the age of 45 years, called the Korean Longitudinal Study of Aging (KLoSA) [17]. The Korea Labor Institute sampled 6171 households from 1000 enumeration districts since 2006 and obtained data on demographics, health status, family structure, income and employment status, and medical history via interviews with follow-up interviews every two years. Five waves are publicly available (2006, 2008, 2010, 2012, and 2014), among which we used the first (2006) and fifth (2014) waves. A total of 966 older women without cognitive impairment (24 points or more of the Korea Mini-mental State Examination, K-MMSE) aged 65 years or older were initially included during the first wave. Individuals without information on BMI or handgrip strength values (*n* = 143) at first wave, who passed away (*n* = 120) and did not respond at the fifth wave (*n* = 118) were excluded. After excluding individuals who lack K-MMSE scores during the fifth wave (*n* = 41), the final study population consisted 544 respondents (Fig. 1).

### Measurement of handgrip strength

Two primary exposure variables were used in this study: handgrip strength and BMI from the first wave. Handgrip strength was measured by using a dynamometer (Model number: NO6103, Manufacturer: TANITA, Japan), which measures handgrip strength in units of 0.1 kgF and has been used in multiple previous studies that determined handgrip strength [18, 19]. Upon measurement, the participant was in a seated or standing position, her elbow at a right angle, and the wrist in neutral position. Two measurements for each hand were taken, with the maximum value from each hand were averaged to determine the final handgrip strength. Handgrip strength was either classified in a binary manner as weak (lower half of handgrip strength, ≤18.5 kgF) or strong (upper half of handgrip strength, > 18.5 kgF), or into tertiles (4.5–17.5 kgF, 18.0–20.5 kgF, and 20.8–27.5 kgF).

### Classification of handgrip strength and BMI

The other primary exposure variable, BMI, was calculated by self-reported data on weight and height during the first wave. As the revised Asia-Pacific BMI criteria by the Western Pacific Region of the World Health Organization classified those with BMI of 25 kg/m^2^ or greater as obese [20], we divided the study population into non-obese (BMI of less than 25 kg/m^2^) and obese (BMI of 25 kg/m^2^ or greater). A previous study investigating the association between BMI calculated by self-reported weight and height data from KLoSA and BMI calculated by measured weight and height on the same study sample (from a sub-sample of 510 individuals from KLoSA) revealed acceptable Pearson’s correlation coefficients (0.865) and specificity (98.0%), but low sensitivity (60.1%) for obesity diagnosis among women [21].

We combined handgrip strength and BMI by first dividing the population into non-obese and obese individuals. Then, the population was further divided according to handgrip strength (lower half and upper half). The population was ultimately divided into: 1) non-obese and weak (lower half) handgrip strength, 2) non-obese and strong (upper half) handgrip strength, 3) obese and weak handgrip strength, and 4) obese and strong handgrip strength.

### Assessment of cognitive impairment and cognitive decline

Incident cognitive impairment was determined by the K-MMSE score on the fifth wave. K-MMSE is used to determine cognitive function by questions designed to assess various categories of cognitive function, such as time and place, orientation, registration, attention and calculation, memory recall, language, and visual construction [22, 23]. The validity of K-MMSE scores has been verified in a previous study [22], in which decreasing scores from a perfect score of 30 indicate declining cognitive function. As K-MMSE scores of 24 or greater are considered normal [22, 23], we defined cognitive impairment as a change of K-MMSE score into 23 or less. Furthermore, we calculated the degree of cognitive function decline by subtracting the K-MMSE scores in 2014 by those in 2006 for each individual.

### Covariates

The covariates considered in this study are age, marital status (married and unmarried), education (elementary, middle school, high school, and college or higher), income (divided into quartiles), insurance (Medicaid and National Health Insurance), area of residence (urban and rural), smoking status (never smoker, ex-smoker, and current smoker), drinking (no and yes), physical activity (self-reported questionnaire of none and at least once per week of exercise), weight change determined by the change in BMI between the fifth and first waves (loss of less than 1 kg/m^2^, no change, and gain of more than 1 kg/m^2^), activities of daily living (0 and 1 or more), depression (the Depression 10-item Scale of 4 or less and more than 4, the Center for Epidemiologic Studies), comorbidity (none among cardiovascular disease, stroke, hypertension, and diabetes, and at least one), and baseline K-MMSE score from the first wave.

### Statistical analysis

The analysis of variance (ANOVA) method for continuous variables and χ^2^ test for categorical variables were performed to compare differences in baseline variables according to BMI and handgrip strength. We first determined the association between baseline handgrip strength and BMI values in the first wave (2006) and cognitive decline in the fifth wave (2014), resulting in an eight-year follow-up period. This was carried out in two ways. We performed logistic regression analyses between the four groups categorized according to BMI and handgrip strength and cognitive decline. Then, the study population was divided into tertiles of increasing handgrip strength and logistic regression analyses was conducted in non-obese and obese groups. The *P* for interaction was calculated to determine whether obesity was a significant factor in the association between handgrip strength and cognitive impairment. Furthermore, the association between baseline handgrip strength and BMI values and degree of cognitive function decline was investigated by linear regression analyses between handgrip strength tertiles and change in K-MMSE scores. Finally, the association between change in handgrip strength and cognitive impairment among those with strong handgrip strength during the first wave was determined by logistic regression analysis to confirm the causality. Among those with strong handgrip strength in the first wave, those with strong handgrip strength (maintained handgrip strength) and weak handgrip strength (weakened handgrip strength) during the fifth wave were compared for development of cognitive decline.

For logistic and linear regression analyses, covariates were adjusted for in a sequential manner: model 1 (age), model 2 (additionally marital status, education, income, insurance, and area of residence), model 3 (additionally smoking status, drinking, and physical activity), and model 4 (additionally activities of daily living, depression, comorbidity, and baseline K-MMSE score). Statistical significance was defined as a *p* value of less than 0.05 in a two-sided manner. All statistical analyses were conducted via Stata version 13.0 (College Station, TX; StataCorp LP).

## Results

### Baseline characteristics

The descriptive characteristics of the study population during the first wave in 2006 are presented in Table 1. The distribution of individuals according to BMI and handgrip strength was 33.1% for non-obese with weak handgrip strength, 39.3% for non-obese with strong handgrip strength, 12.5% for obese with weak handgrip strength and 15.1% for obese with strong handgrip strength. Among the four groups divided by BMI and handgrip strength, those with strong handgrip strength were younger compared to those with weak handgrip strength. Non-obese and obese women with strong handgrip were younger than those with weak handgrip strength. Mean ages were 68.7 and 68.8 years in non-obese and obese women with strong handgrip strength, while 71.4 and 70.6 years in non-obese and obese women with weak handgrip strength, respectively. Obese women with strong handgrip strength were likely to be less depressive and more physically active than obese women with weak handgrip strength. There was no significant difference in baseline K-MMSE score at baseline according to the handgrip strength and obesity. Furthermore, a sensitivity analysis of the characteristics between study participants and non-participants are shown in (Additional file 1: Table S1) There was a significance difference in age, area of residence, activities of daily living, and baseline K-MMSE score among participants and non-participants (all *p* values < 0.05).

**Table 1:** Descriptive characteristics of study participants divided by BMI and handgrip strength

BMI	Non-obese ^a^		Obese ^a^		*p* value
Handgrip Strength ^b^	Weak	Strong	Weak	Strong	
Number of people	180	214	68	82	
Proportion, %	33.1	39.3	12.5	15.1	
Age, years					
Mean	71.4	68.7	70.6	68.8	< 0.001
SD	4.8	3.9	4.3	3.6	
Marital status, %					
Married	31.1	44.0	10.4	14.5	0.209
Unmarried	34.7	35.6	14.2	15.5	
Education, %					
Elementary	32.0	40.7	12.9	14.4	0.351
Middle	37.3	30.5	10.2	22.0	
High	40.7	32.2	11.9	15.3	
College or higher	18.8	62.5	12.5	6.3	
Income, %					
1st quartile	34.7	33.3	16.9	15.1	0.060
2nd quartile	34.7	45.2	8.1	12.1	
3rd quartile	34.9	45.8	4.8	14.5	
4th quartile	27.1	41.7	10.4	20.8	
Insurance, %					
Medicaid	50.0	18.4	21.1	10.5	0.012
NHI	31.8	40.9	11.9	15.4	
Area of residence, %					
Urban	32.7	37.1	13.7	16.6	0.087
Rural	34.3	46.3	9.0	10.5	
Smoking status, %					
Never smoker	35.0	39.5	12.8	14.7	0.561
Ex-smoker	25.0	25.0	0.0	50.0	
Current smoker	37.5	37.5	6.3	18.8	
Drinking, %					
No	34.8	36.6	12.8	15.8	0.002
Yes	19.0	62.1	10.3	8.6	
Physical activity, %					
None	31.1	39.0	14.6	15.2	0.258
1 or more/week.	36.1	39.8	9.3	14.8	
Weight change, %					
Loss	49.1	34.0	11.3	5.7	0.025
No change	31.0	40.2	12.2	16.6	
Gain	43.8	31.3	25.0	0.0	
ADL, %					
0	33.0	39.3	12.6	15.1	0.938
1 or more	35.7	42.9	7.1	14.3	
Depression, %					
No	28.0	43.7	10.5	17.8	0.001
Yes	41.4	32.3	15.2	11.0	
Comorbidity, %					
0	31.3	43.5	11.8	13.4	0.273
1 or more	34.8	35.5	13.1	16.7	
Baseline K-MMSE					
Mean	26.3	26.7	26.5	26.8	0.072
SD	1.7	1.7	1.6	1.6	

^a^non-obese, BMI < 25 kg/m^2^; obese, BMI **≥** 25 kg/m^2^

^b^Handgrip strength: weak, lower half, < 18.5 kgF; strong, upper half, ≥18.5 kgF

Acronyms: *BMI* body mass index, *K-MMSE* Korea Mini-mental state examination, *SD* standard deviation, *NHI* National Health Insurance, *ADL* activities of daily living

### Association between handgrip strength with BMI and development of cognitive impairment

The association between handgrip strength with BMI and cognitive decline is depicted in Table 2. There was a significant interaction between obesity and handgrip strength (*p* value = 0.013 in model 4) (see Additional file 2). Strong handgrip strength was associated with reduced likelihood of developing cognitive decline compared to weak handgrip strength in obese women (adjusted odds ratio, aOR 0.23, 95% confidence interval, CI 0.08–0.66) (Table 2). Strong handgrip strength in non-obese women did not show the significant association with cognitive decline (aOR 1.26, 95% CI 0.75–2.13).

**Table 2:** Associations between handgrip strength with BMI and cognitive decline

BMI ^a^	Non-obese		Obese		*P*_interaction_
Handgrip Strength ^b^	Weak	Strong	Weak	Strong	
Number of cases	94	105	39	32	
Model 1	1.00 (reference)	1.15 (0.71–1.84)	1.00 (reference)	0.66 (0.32–1.38)	0.065
Model 2	1.00 (reference)	1.04 (0.63–1.73)	1.00 (reference)	0.39 (0.17–0.92)	0.029
Model 3	1.00 (reference)	1.13 (0.67–1.88)	1.00 (reference)	0.28 (0.11–0.72)	0.025
Model 4	1.00 (reference)	1.26 (0.75–2.13)	1.00 (reference)	0.23 (0.08–0.66)	0.013

^a^non-obese, BMI < 25 kg/m^2^; obese, BMI **≥** 25 kg/m^2^

^b^Handgrip strength: weak, lower half, < 18.5 kgF; strong, upper half, ≥18.5 kgF

Model 1: odds ratio by logistic regression analysis adjusted for age (95% confidence interval)

Model 2: additionally adjusted for marital status, education, income, insurance, and area of residence

Model 3: additionally adjusted for smoking status, drinking, physical activity, and weight change

Model 4: additionally adjusted for activities of daily living, depression, comorbidity, and baseline K-MMSE score

*P* for interaction of BMI and handgrip strength for cognitive impairment

Acronyms: BMI, body mass index

### Association between handgrip tertiles according to BMI and cognitive impairment

The risk for cognitive impairment was not significantly different according to tertiles of handgrip strength in the total study population (Table 3). However, among obese women, those with the highest tertile of handgrip strength had reduced likelihood of developing cognitive impairment compared to the lowest tertile of handgrip strength (aOR 0.16, 95% CI 0.04–0.70). Furthermore, there was a significant linear trend between handgrip strength and the risk of cognitive decline among obese women (*p* for trend 0.016). Such associations were not observed in non-obese women.

**Table 3:** Associations between handgrip strength with BMI and cognitive decline according to handgrip strength tertiles and obesity

Handgrip Strength ^a^	1st tertile	2nd tertile	3rd tertile	*p* for trend
BMI combined				
Number of cases	104	93	73	
Model 1	1.00 (reference)	1.04 (0.67–1.61)	0.90 (0.55–1.47)	0.684
Model 2	1.00 (reference)	1.05 (0.64–1.71)	0.75 (0.44–1.28)	0.303
Model 3	1.00 (reference)	1.08 (0.66–1.77)	0.77 (0.45–1.33)	0.361
Model 4	1.00 (reference)	1.12 (0.67–1.87)	0.88 (0.50–1.53)	0.649
Non-obese ^b^				
Number of cases	70	75	53	
Model 1	1.00 (reference)	1.31 (0.77–2.23)	1.13 (0.61–2.07)	0.669
Model 2	1.00 (reference)	1.27 (0.70–2.30)	1.09 (0.57–2.11)	0.752
Model 3	1.00 (reference)	1.30 (0.71–2.39)	1.16 (0.59–2.26)	0.633
Model 4	1.00 (reference)	1.45 (0.77–2.72)	1.42 (0.72–2.79)	0.296
Obese ^b^				
Number of cases	34	18	20	
Model 1	1.00 (reference)	0.53 (0.23–1.23)	0.56 (0.23–1.34)	0.205
Model 2	1.00 (reference)	0.51 (0.18–1.40)	0.29 (0.10–0.86)	0.027
Model 3	1.00 (reference)	0.42 (0.14–1.28)	0.19 (0.05–0.68)	0.011
Model 4	1.00 (reference)	0.35 (0.10–1.17)	0.16 (0.04–0.70)	0.016

^a^Handgrip strength: 1st tertile (≤17.5 kgF), 2nd tertile (18.0 to 20.5 kgF), and 3rd tertile (≥20.8 kgF)

^b^non-obese, BMI < 25 kg/m^2^; obese, BMI **≥** 25 kg/m^2^

Model 1: odds ratio by logistic regression analysis adjusted for age (95% confidence interval)

Model 2: additionally adjusted for marital status, education, income, insurance, and area of residence

Model 3: additionally adjusted for smoking status, drinking, physical activity, and weight change

Model 4: additionally adjusted for activities of daily living, depression, comorbidity, and baseline K-MMSE score

Acronyms: BMI, body mass index

### Association between handgrip strength with BMI and change in K-MMSE score

**Fig. 2:**
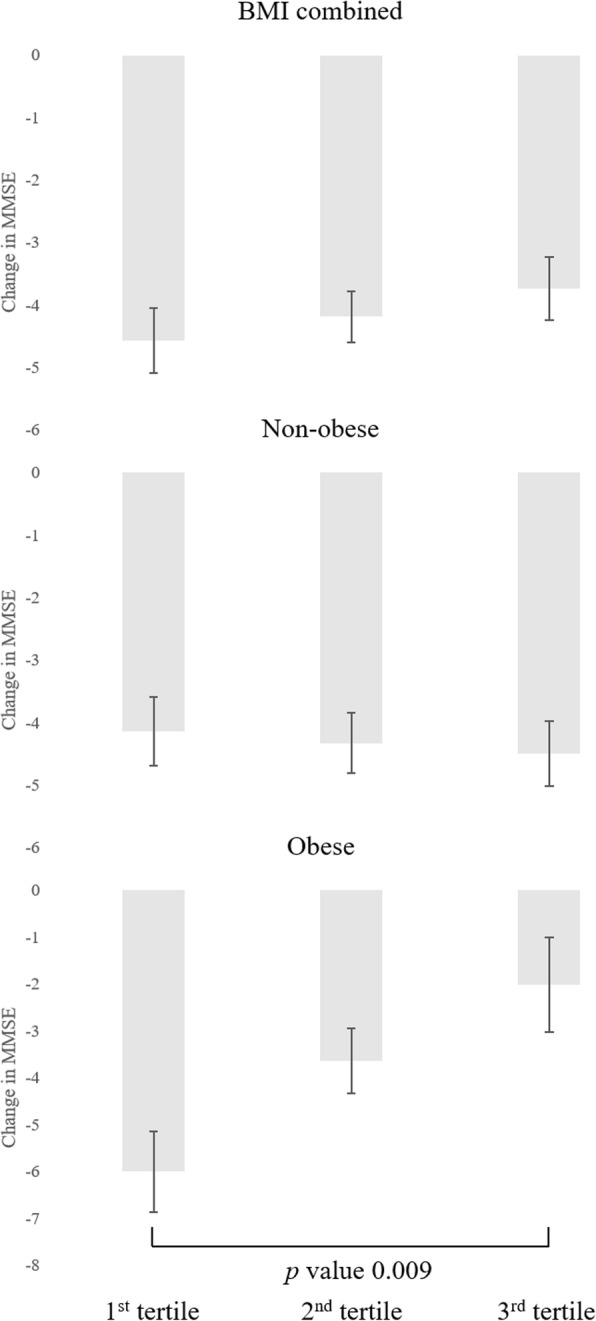
Adjusted means of change in K-MMSE scores according to handgrip tertiles and obesity. Non-obese, BMI < 25 kg/m^2^; obese, BMI **≥** 25 kg/m^2.^ Handgrip strength: 1st tertile (≤17.5 kgF), 2nd tertile (18.0 to 20.5 kgF), and 3rd tertile (≥20.8 kgF). Adjusted mean values adjusted for age, marital status, education, income, insurance, area of residence smoking status, drinking, physical activity, activities of daily living, depression, comorbidity, and baseline K-MMSE score, *p*-value calculated by linear regression analysis. Black bar indicates standard error. Acronyms: BMI, body mass index

The association between handgrip strength with BMI and the degree of cognitive function decline calculated by difference in K-MMSE scores were presented in Fig. 2. The highest tertile handgrip strength was significantly associated with smaller declines in K-MMSE scores compared to the lowest tertile handgrip strength in obese women (*p* value 0.009), while the highest tertile of handgrip strength did not in non-obese women. Furthermore, there was a notable linear trend between the degree of cognitive function decline and handgrip strength among obese women (*p* for trend 0.048).

### Change in handgrip strength and cognitive impairment

We evaluated the association weakened handgrip strength and cognitive impairment at fifth wave among those who had strong handgrip strength at first wave (*n* = 137) regardless of obesity (Table 4). Weakened handgrip strength was associated with increased risk of cognitive impairment (aOR 2.28, 95% CI 1.23–4.24) compared to maintained handgrip strength.

**Table 4:** Associations between change in handgrip strength and cognitive decline among those with strong handgrip strength

Change in Handgrip Strength ^a^	Maintained	Weakened
Number of cases	68	69
Model 1	1.00 (reference)	2.15 (1.29–3.61)
Model 2	1.00 (reference)	2.13 (1.19–3.81)
Model 3	1.00 (reference)	2.07 (1.13–3.79)
Model 4	1.00 (reference)	2.28 (1.23–4.24)

^a^Change in handgrip strength: maintained, strong handgrip strength in fifth wave; weakened, weak handgrip strength in fifth wave

Model 1: odds ratio by logistic regression analysis adjusted for age (95% confidence interval)

Model 2: additionally adjusted for marital status, education, income, insurance, and area of residence

Model 3: additionally adjusted for smoking status, drinking, physical activity, and weight change

Model 4: additionally adjusted for activities of daily living, depression, comorbidity, and baseline K-MMSE score

## Discussion

In this longitudinal study, we have demonstrated that strong handgrip strength was associated with lower risk of cognitive impairment compared to weak handgrip strength among obese women. Meanwhile, strong handgrip strength was not associated with lower risk of cognitive impairment in non-obese women. Moreover, decline in handgrip strength was significantly associated with cognitive impairment.

Our results are in agreement with previous studies that have shown that strong handgrip strength was protectively associated with cognitive decline [12, 13]. In a cross-sectional study, mild cognitive impairment was associated with weak handgrip strength [12]. A longitudinal study among 2160 Mexican Americans aged over 65 years have also shown that the highest handgrip strength quartile was associated with better cognitive function over 7 years [13]. A study from the Rush Memory and Aging Project has also reported that strong muscle strength was associated with decreased risk of Alzheimer’s disease and mild cognitive impairment [24]. The Rush Memory and Aging Project assessed comprehensive muscle strength with measurement of nine muscle groups including handgrip strength. However, both longitudinal studies adjusted for BMI as a covariate and did not stratify the subjects according to obesity.

Several possible mechanisms may explain the association between handgrip strength and cognitive function. First, weak handgrip strength may be an early sign of cognitive impairment, as handgrip strength could be reflected by change of nervous system activity or white matter integrity [25]. A prospective study among 555 subjects aged 85 years at baseline revealed that better cognitive performance for attention and processing speed was significantly associated with both stronger and slower decline in handgrip strength [11]. The authors suggested that cognitive function may precede muscle weakness, which could be explained by the fact that strong handgrip strength needs better neuromuscular coordination under well-operated executive functions, especially in the frontal lobe [25]. In our study, weakened handgrip strength was associated with cognitive impairment compared to sustained strong handgrip strength, suggesting that weakened handgrip strength could precede cognitive impairment. Therefore, handgrip strength could be a simple and useful marker in predicting future cognitive impairment.

Second, weak muscle strength and cognitive impairment may share common pathophysiological pathways such as systemic inflammation, insulin resistance and oxidative stress, all of which may contribute to both weak muscle strength and cognitive impairment [26, 27]. Furthermore, factor related to sociodemographics [28], depressive symptoms, health behavior or nutritional state may also affect the risk of cognitive impairment according to muscle strength. Specifically, increased physical activity is highly associated with handgrip strength [29], while also attenuating cognitive decline by the promotion of increased neuroplasty and cerebral blood flow [30]. Previous studies have shown that under-nutrition is associated with weak handgrip strength as well as cognitive impairment in older adults [31].

Our study showed that strong handgrip strength was associated with lower risk of cognitive impairment in obese women, but not in non-obese women. This discrepancy in association of handgrip strength and cognitive impairment according to obesity could be explained by differences of body composition [32]. Participants with high BMI who have strong handgrip strength may have greater muscle mass and less fat mass compared to those with weak handgrip strength. On the other hand, obese participants with weak handgrip strength may have low muscle mass and high fat mass, a state referred to sarcopenic obesity. Excess body fat has previously been shown to be associated with brain atrophy [33] and reduction of hippocampal function through alteration in deoxyribonucleic acid methylation of memory-associated genes [34]. Sarcopenia may also aggravate the risk of cognitive impairment via decreased secretion of cytokines such as insulin-like growth factors [35].

Several limitations must be considered upon interpreting out results. First, the number of subjects was relatively small and may thus lack sufficient statistical power. Our results cannot be generalized to men as the study population was limited to women. Second, selection bias of the study population may exist considering of different baseline characteristics between participants and non-participants. People with poor health conditions are more likely to have passed away or not respond during the fifth wave. Third, BMI was measured using self-reported height and weight. Particularly, as obesity determined by self-reported values yielded high specificity but low sensitivity, there may have been an underestimation of obese individuals. In a previous study using the National Health and Nutrition Examination Survey, there was an average − 0.67 (SD, 0.04) kg/m2 in difference between self-reported and measured BMI values in women. However, there were no difference in the association between self-reported BMI-defined obesity and obesity-related markers compared to association between measured BMI-defined obesity and obesity-related markers, suggesting that self-reported BMI is sufficiently acceptable for epidemiological studies [36]. In addition, we could consider applying high BMI threshold for obesity in older adults because of the low risk of cognitive decline [24] and mortality [37] in overweight groups or due to height reduction [38]. Moreover, other measures of body composition, particularly the proportions of fat and muscle mass, would be useful in further investigating the association between obesity and cognitive function.

Fourth, although the modality of exercise may be an important factor in the association between handgrip strength and cognitive function, we could not adjust for this potential confounder due to the lack of information. Fifth, while MMSE is a widely used cognitive test, it is insensitive detecting early stages of cognitive decline [39], and thus other measurements of cognitive impairment in future studies would be beneficial. Sixth, although measurement of handgrip strength is a useful and simple method to assess upper extremity muscle strength, we could not reflect other aspects of muscle strength, such as quadriceps strength [40] due to the lack of data. Seventh, confounding factors that affect handgrip strength over time such as nutritional state or presence of arthritis in hand could not be considered [41]. Finally, handgrip strength was determined by the maximum value of two measurements rather than three. Future studies using handgrip strength measured by the best of three measurements are needed.

Despite these limitations, to our knowledge, this is first study to elucidate the association between handgrip strength and cognitive function stratified by obesity. In addition, we used a national representative survey. KLoSA is conducted by the Ministry of Labor of Korea from 2006 enrolled participants who randomly selected by a multistage, stratified probability sampling among of community-dwelling Koreans aged ≥45 years after consideration of age, sex, and geographic area [42].

## Conclusions

In conclusion, strong handgrip strength in obese women was associated with reduced risk of cognitive impairment. Handgrip strength may be a simple and useful marker for predicting future cognitive impairment among obese women. Further research is needed to determine why the relationship between grip strength and cognitive function was more prominent in obese groups.

## Additional files

Additional file 1: **Table S1.** Sensitivity analysis of the descriptive characteristics between study participants with non-participants. (DOCX 16 kb)

Additional file 2: **Figure S1.** Predictive margins for cognitive impairment according to handgrip strength and obesity. Predictive margins calculated by logistic regression analysis after adjustments for age, marital status, education, income, insurance, area of residence, smoking status, drinking, physical activity, weight change, activities of daily living, depression, comorbidity, and baseline K-MMSE score. Obesity: non-obese, BMI < 25 kg/m^2^; obese, BMI ≥ 25 kg/m^2^. Handgrip strength: weak, lower half, < 18.5 kgF; strong, upper half, ≥18.5 kgF. (PNG 27 kb)

## Abbreviations

BMI: Body mass index
KLoSA: Korean Longitudinal Study of Aging
K-MMSE: Korean Mini-Mental State Examination
