# Supplementary material for: Association among handgrip strength, body mass index and decline in cognitive function among the elderly women
Source: BMC Geriatr. 2018 Sep 24;18:225. doi: 10.1186/s12877-018-0918-9 (PMC6154935; doi:10.1186/s12877-018-0918-9)
Supplement: Supplementary file 1 — Table S1. Sensitivity analysis of the descriptive characteristics between study participants with non-participants. (DOCX 16 kb) [file 12877_2018_918_MOESM1_ESM.docx]

**Supplemental Table 1.** Sensitivity analysis of the descriptive characteristics between study participants with non-participants.

| Population | Participants | Non-participants | *p* value |
| --- | --- | --- | --- |
| Number of people | 544 | 422 |  |
| Age, years |  |  |  |
| Mean | 69.8 | 73.3 | <0.001 |
| SD | 4.4 | 6.8 |  |
| Marital status |  |  |  |
| Married | 44.6 | 40.4 | 0.409 |
| Unmarried | 55.4 | 59.7 |  |
| Education |  |  |  |
| Elementary | 75.1 | 73.0 | 0.828 |
| Middle | 10.9 | 12.1 |  |
| High | 11.1 | 12.3 |  |
| College or higher | 3.0 | 2.6 |  |
| Income |  |  |  |
| 1^st^ quartile | 24.3 | 27.6 | 0.483 |
| 2^nd^ quartile | 26.0 | 25.9 |  |
| 3^rd^ quartile | 25.2 | 20.9 |  |
| 4^th^ quartile | 24.5 | 25.6 |  |
| Insurance |  |  |  |
| Medicaid | 7.0 | 6.0 | 0.515 |
| NHI | 93.0 | 94.1 |  |
| Area of residence |  |  |  |
| Urban | 75.3 | 85.6 | <0.001 |
| Rural | 24.7 | 14.4 |  |
| Smoking status |  |  |  |
| Never smoker | 96.3 | 96.0 | 0.932 |
| Ex-smoker | 0.7 | 1.0 |  |
| Current smoker | 3.0 | 3.1 |  |
| Drinking |  |  |  |
| No | 89.3 | 89.6 | 0.888 |
| Yes | 10.7 | 10.4 |  |
| Physical activity |  |  |  |
| None | 59.9 | 65.3 | 0.086 |
| 1 or more/week | 40.2 | 34.8 |  |
| Weight change |  |  |  |
| Loss | 9.8 | 9.2 | 0.956 |
| No change | 87.3 | 87.7 |  |
| Gain | 3.0 | 3.1 |  |
| ADL |  |  |  |
| 0 | 97.6 | 87.7 | <0.001 |
| 1 or more | 2.4 | 12.3 |  |
| Depression |  |  |  |
| No | 61.0 | 56.1 | 0.128 |
| Yes | 39.0 | 43.9 |  |
| Comorbidity |  |  |  |
| 0 | 48.3 | 43.7 | 0.163 |
| 1 or more | 51.8 | 56.3 |  |
| Baseline K-MMSE |  |  |  |
| Mean | 26.6 | 26.3 | 0.030 |
| SD | 1.7 | 1.7 |  |

Acronyms: SD, standard deviation; NHI, National Health Insurance; ADL, activities of daily living; K-MMSE, Korea mini-mental state examination.
