# Supplementary figures and images for: Association among handgrip strength, body mass index and decline in cognitive function among the elderly women
Source: BMC Geriatr. 2018 Sep 24;18:225. doi: 10.1186/s12877-018-0918-9 (PMC6154935; doi:10.1186/s12877-018-0918-9)

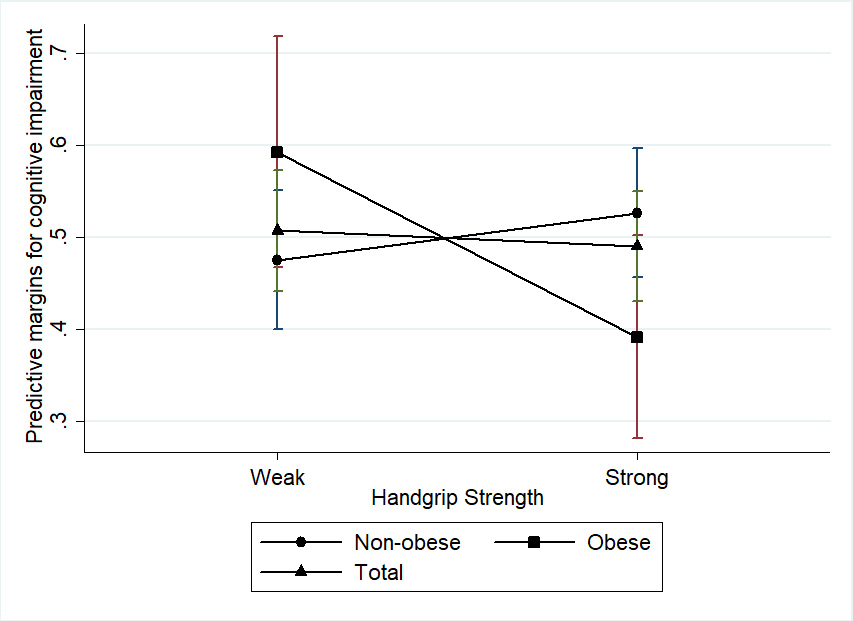

Supplement: Supplementary file 2 — Figure S1. Predictive margins for cognitive impairment according to handgrip strength and obesity. Predictive margins calculated by logistic regression analysis after adjustments for age, marital status, education, income, insurance, area of residence, smoking status, drinking, physical activity, weight change, activities of daily living, depression, comorbidity, and baseline K-MMSE score. Obesity: non-obese, BMI < 25 kg/m2; obese, BMI ≥ 25 kg/m2. Handgrip strength: weak, lower half, < 18.5 kgF; strong, upper half, ≥18.5 kgF. (PNG 27 kb) [file 12877_2018_918_MOESM2_ESM.png]
